# Age-dependent changes in Ca^2+^ homeostasis in peripheral neurones: implications for changes in function

**DOI:** 10.1111/j.1474-9726.2007.00298.x

**Published:** 2007-06

**Authors:** John N Buchholz, Erik J Behringer, William J Pottorf, William J Pearce, Conwin K Vanterpool

**Affiliations:** 1 Department of Physiology and Pharmacology, Loma Linda University, School of Medicine Loma Linda, CA 92350, USA; 2 Department Scientific Affairs, Astra Zeneca 1800 Concord Pike Wilmington DE 19850, USA; 3 Department of Biological Sciences, Carter Hall, Alabama A & M 4900 Meridian Street, Normal, AL 35762, USA

**Keywords:** age and calcium homeostasis, calcium buffering, superior cervical ganglion

## Abstract

Calcium ions represent universal second messengers within neuronal cells integrating multiple cellular functions, such as release of neurotransmitters, gene expression, proliferation, excitability, and regulation of cell death or apoptotic pathways. The magnitude, duration and shape of stimulation-evoked intracellular calcium ([Ca^2+^]i) transients are determined by a complex interplay of mechanisms that modulate stimulation-evoked rises in [Ca^2+^]i that occur with normal neuronal function. Disruption of any of these mechanisms may have implications for the function and health of peripheral neurones during the aging process. This review focuses on the impact of advancing age on the overall function of peripheral adrenergic neurones and how these changes in function may be linked to age-related changes in modulation of [Ca^2+^]i regulation. The data in this review suggest that normal aging in peripheral autonomic neurones is a subtle process and does not always result in dramatic deterioration in their function. We present studies that support the idea that in order to maintain cell viability peripheral neurones are able to compensate for an age-related decline in the function of at least one of the neuronal calcium-buffering systems, smooth endoplasmic reticulum calcium ATPases, by increased function of other calcium-buffering systems, namely, the mitochondria and plasmalemma calcium extrusion. Increased mitochondrial calcium uptake may represent a ‘weak point’ in cellular compensation as this over time may contribute to cell death. In addition, we present more recent studies on [Ca^2+^]i regulation in the form of the modulation of release of calcium from smooth endoplasmic reticulum calcium stores. These studies suggest that the contribution of the release of calcium from smooth endoplasmic reticulum calcium stores is altered with age through a combination of altered ryanodine receptor levels and modulation of these receptors by neuronal nitric oxide containing neurones.

## Introduction

Calcium is widely recognized as a universal second messenger within neuronal cells and integrates multiple cellular functions. These include release of neurotransmitters, gene expression, proliferation, excitability and regulation of cell death or apoptotic pathways (Malenka *et al*., 1989; Choi, 1992; Berridge, 1995, 1998; Clapham, 1995; Ginty, 1997; Wuytack *et al*., 2002).

At rest, neurones maintain a large intracellular calcium ([Ca^2+^]i) concentration gradient between the extracellular milieu and cytosol. In peripheral neurones, calcium signaling begins with the opening of L and N and some R type calcium channels, allowing calcium to flow from outside of the cell into the cytosol (Kostyuk, 1989; Trouslard *et al*., 1993; Vanterpool *et al*., 2005). Much of this signal is damped by calcium-buffering proteins. However, calcium signaling initiated by calcium influx is sustained by the rapid release of calcium from smooth endoplasmic reticulum (SER) calcium stores. This process is known as calcium-induced calcium release (CICR) and is mediated by calcium acting on ryanodine receptor (RyR) channels (Belan *et al*., 1993; Verkhratsky & Shmigol, 1996; Usachev & Thayer, 1997, 1999a,b; Verkhratsky & Petersen, 1998; Akita & Kuba, 2000).

**Fig. 1:**
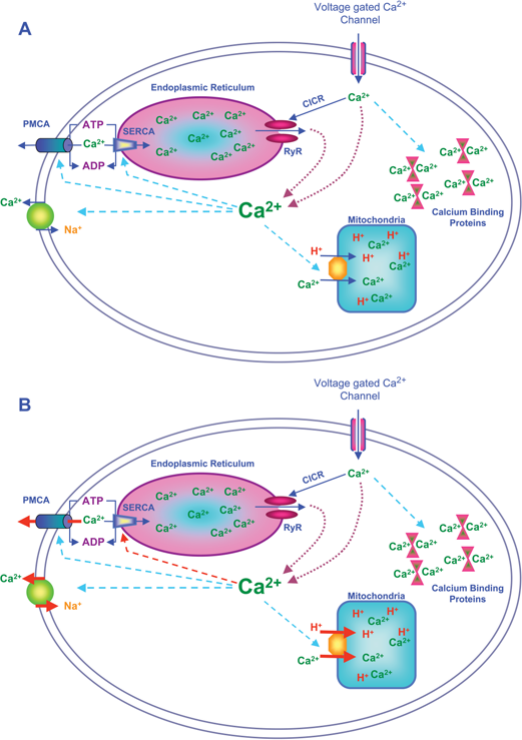
(A) Representation of mechanisms that modulate stimulation-evoked [Ca^2+^]i transients in peripheral adrenergic nerves. Depolarization increases [Ca^2+^]i by rapid calcium influx through voltage-gated calcium channels. Calcium is rapidly attenuated via calcium-binding proteins and the residual calcium signal acting on ryanodine receptors (RyR) evoke release of calcium from the endoplasmic reticulum known as calcium-induced calcium release (CICR). The elevation in [Ca^2+^]i is controlled by a dynamic interplay of buffering systems: (1) smooth endoplasmic reticulum calcium ATPases (SERCA) sequestration into the ER that serves to buffer and refill ER calcium stores thus maintaining the ability of the neurone to undergo repetitive CICR; (2) mitochondrial calcium uptake by a H^+^/Ca^2+^ uniporter; (3) removal of calcium via plasma membrane calcium ATPases (PMCA) pumps and the Na^+^/Ca^2+^ exchanger. Plum dotted lines represent calcium influx and release pathways that elevate [Ca^2+^]i. Light blue dashed lines represent calcium-buffering pathways that control increases in [Ca^2+^]i and restoration to resting levels. (B) Model illustrating the hypothesis that advancing age in the absence of pathology, results in a subtle decline in the control of [Ca^2+^]i. Compensation by other control mechanisms may allow neurones to adapt to an age-related decline in control of [Ca^2+^]i. Specifically, this model illustrates the mechanisms that lead to elevated [Ca^2+^]i in aged peripheral adrenergic neurones. The rise in [Ca^2+^]i mediated via calcium influx and release from the SER is buffered by SERCA whose function declines with age (broken red line). In response to the decline in SERCA function mitochondria, PMCA and Na^+^/Ca^2+^ exchanger compensate (thick red solid lines) for the decline in SERCA function by increasing Ca^2+^ uptake and removal so as to preserve overall neuronal viability. In addition, the decline in SERCA function may possibly alter ER Ca^2+^ filling levels, which may have consequences for sustained CICR in senescent neurones.

Following a rapid rise in [Ca^2+^]i, and depending on the magnitude of the [Ca^2+^]i transient, complex buffering systems that include multiple calcium-buffering proteins, smooth endoplasmic reticulum calcium ATPases (SERCA), mitochondrial calcium uptake, plasmalemma calcium ATPases (PMCA) and the sodium–calcium exchanger (Na^+^/Ca^2+^), work together to reduce [Ca^2+^]i from its peak levels, and restore [Ca^2+^]i back to resting levels (Werth & Thayer, 1994; Buchholz *et al*., 1996; Werth *et al*., 1996; Usachev & Thayer, 1999a; Pottorf *et al*., 2000a,c, 2002; Wuytack *et al*., 2002). In particular, the CICR process is dependent on buffering by SERCA as this buffering not only participates in control of [Ca^2+^]i levels, but serves as a refilling of endoplasmic reticulum calcium stores (Vanterpool *et al*., 2005). These complex processes are illustrated in Fig. 1(A). Overall, the magnitude, duration, and shape of stimulation-evoked [Ca^2+^]i transients are determined by a complex interplay of mechanisms that increase, buffer, and return these transients to resting levels. Disruption of any of these mechanisms may have implications for the function and health of peripheral neurones during the aging process. This review will focus on the impact of advancing age on the overall function of peripheral adrenergic neurones and how these changes in function may be linked to age-related changes in modulation of [Ca^2+^]i levels. In addition, other consequences of age-related alterations in the modulation of [Ca^2+^]i in peripheral adrenergic neurones will be addressed. Data from studies on peripheral sensory and central neurones will also be incorporated into the discussion, as these models lend insight into the function of adrenergic neurones during the aging process.

## Why are adrenergic nerves arising from the superior cervical ganglion an important study model?

The superior cervical ganglion (SCG) has been suggested to be a peripheral neuroendocrine center because of the plethora of tissues that receive adrenergic input from this source (Cardinali *et al*., 1981). The SCG has been shown to be an important modulator of cardiac function that relays cardioregulatory impulses from the central nervous system (CNS) to the SCG axons terminating in the heart that release noradrenaline (NA) and activate β1 adrenergic receptors causing strengthening of the heart contractions (Wingerd *et al*., 2004). In addition, altered adrenergic innervation to the heart has been implicated in sudden cardiac death following myocardial infarction (Chen *et al*., 2001). Furthermore, the maintenance of neuronal numbers in the SCG by trophic factors such as nerve growth factor (NGF) is necessary to ensure proper function of the target tissues innervated by the SCG. For example, the levels of NGF decline with advancing age, however, the trophic response of perivascular sympathetic nerves to NGF is preserved (Isaacson & Crutcher, 1998; Dickason & Isaacson, 2002). Thus, the response to NGF during the aging process may have implications for the function of target organs innervated by the SCG (reviewed by Cowen & Gavazzi, 1998). Overall, the evidence in terms of normal aging argues that peripheral neurones attempt to maintain homeostasis and can respond to trophic influences later in life.

In the rat cerebrovasculature, adrenergic and sensory innervation is fully developed within the first 30 days of life (Tsai *et al*., 1989). Specifically, in the cerebrovasculature adrenergic innervation influences the development of cerebral blood vessels and their motor function, as their presence is necessary for angiogenesis and the modulation of contractile function. For example, in rabbits aged 3–20 weeks, removal of the SCG results in loss of vascular smooth muscle mass, reduced wall thickness and attenuated contractility (Bevan & Tsuru, 1981).

In addition to the cardiovascular regulatory effects of the SCG, it also plays a role in protecting the cerebrovasculature from stroke. Hemorrhagic stroke accounts for about 10% of cerebrovascular disease with a peak incidence near the age of 60 years and high probability of morbidity or mortality. Risk factors for stroke include age-related hypertension and changes in the structure of cerebral blood vessels (Abbott *et al*., 2003; Zhang *et al*., 2003; Lawes *et al*., 2004). Numerous mechanisms contribute to regulation of cerebral blood flow and modulate blood vessel wall tension (Faraci & Heistad, 1998; Zhang *et al*., 2003). Spontaneous constriction of blood vessels in response to increased blood pressure from 60 to 140 mmHg is called myogenic tone or autoregulation, which reduces wall tension and risk of blood vessel rupture. At systolic pressures, above 140 mmHg myogenic response alone can no longer control wall tension, and activation of adrenergic nerves arising from the SCG provides additional constrictor response to protect the cerebrovasculature from ruptured blood vessels (Van Riper & Bevan, 1991; Faraci & Heisted, 1998; Furuichi *et al*., 1999). More recent studies on how neural input regulates cerebrovascular tone and blood flow have resulted in the rediscovery of the ‘neurovascular unit’. These studies suggest that the combination of neuronal input into smooth muscle and the inherent contractile properties of smooth muscle provide for the optimum function of cerebral blood vessels (reviewed by Hamel, 2006). Given that adrenergic nerves arising from the SCG innervate numerous organs including the cerebrovasculature and the importance of their homeostatic modulatory function, our group has chosen to focus our aging studies on this neuronal model.

## Overview of aging and calcium regulation in neurones

Aging in all creatures is inexorable and has been suggested to be a combination of developmental changes, genetic defects, environmental influences, and an inborn genetic aging process (Harman, 1998; Guarente & Kenyon, 2000; Clancy *et al*., 2001; Tatar *et al*., 2001; Troen, 2003). These studies suggest that basic metabolism and ability to control accumulation of oxidants as well as genetic control pathways are involved in the aging process. For example, caloric restriction prolongs lifespan and reduces age-related morbidity and organ pathology (Bodkin *et al*., 2003; Forster *et al*., 2003). Overall, studies on caloric restriction, reduced oxidative stress, and lifespan render little explanation of age-related changes in the function of critical organ and neuronal systems, and little is known about the vulnerability of particular physiological processes to advancing age.

Given that calcium acts as a universal second messenger in neurones, subtle age-related declines in mechanisms that modulate stimulation-evoked increases in [Ca^2+^]i have been hypothesized to contribute to age-related neuronal dysfunction and degeneration (Kirischuk & Verkhratsky, 1996; Verkhratsky & Toescu, 1998). In particular, one potential mechanism of calcium-mediated cell death suggests that calcium overload results in mitochondrial dysfunction leading to mitochondrial calcium overload and activation of caspases that mediate cell apoptosis (Ichas & Mazat, 1998; Thibault *et al*., 1998; Begley *et al*., 1999).

An important issue in aging studies is a tendency to assume a general age-related deterioration of calcium regulatory processes leading to increased susceptibility to pathology and cell death (Porter *et al*., 1997). This assumption does not take into account compensatory mechanisms that serve to regulate [Ca^2+^]i homeostasis, thus maintaining some degree of neuronal function in senescent neurones or during acute insults such as stroke (Murchinson & Griffith, 1998; Verkhratsky & Toescu, 1998; Lee *et al*., 1999; Griffith *et al*., 2000; Pottorf *et al*., 2000a, 2002). Studying normal aging uncomplicated by disease holds the most promise in trying to understand the aging process under normal circumstances.

## Aging and alterations in control of neurotransmitter release in adrenergic neurones: multiple mechanisms

The risk of stroke increases with age, and the single most important factor is rising systolic blood pressure (Faraci & Heisted, 1998; Abbott *et al*., 2003; Zhang *et al*., 2003; Lawes *et al*., 2004). Systolic blood pressure rises in both F344-Rats and in humans and is associated with increased plasma catecholamine levels suggesting that there is a fundamental age-related change in the function of peripheral adrenergic nerves (Palmer *et al*., 1978; Esler *et al*., 1981; Barnes *et al*., 1982; Yu *et al*., 1985; Insel, 1993). This concept is supported by studies showing that stimulation-evoked fractional NA release increases with age in arteries studied *in vitro*. These include the rat tail artery and superior mesenteric artery (Buchholz & Duckles, 1990; Buchholz *et al*., 1998). In addition, we have shown that the age-related increase in NA release is not dependent on any particular stimulation frequency as the increase occurs over a wide range of frequencies (Tsai *et al*., 1995). The change in function of vascular adrenergic neurones may be explained by multiple mechanisms. For example, changes in density of adrenergic neurones, NA content, re-uptake, function of prejunctional inhibitory α_2_-autoreceptors, and calcium regulation (Pottorf *et al*., 2000b). Furthermore, additional complicating factors must also be addressed. Our studies and others have shown that the SCG (Dun *et al*., 1995; Wu *et al*., 1997) and the cerebral vasculature contain adrenergic and neuronal nitric oxide (nNOS) containing nerves; NO released from nNOS neurones augments stimulation-evoked NA release in both blood vessels and the CNS (Montague *et al*., 1994; Yamamoto *et al*., 1997; Zhang *et al*., 1998; Lee *et al*., 2000; Mbaku *et al*., 2000, 2003). Facilitation of the function of adrenergic nerves via nNOS nerves may occur through enhancement of Ca^2+^ influx and/or Ca^2+^ release from internal stores. These mechanisms may also be altered with age and is the subject of early ongoing studies in our laboratory.

### NA content and adrenergic density in the periphery

The content of NA in peripheral organs and blood vessels has been used as an index of adrenergic density. In the rat heart, NA content decreases with age (Martinez *et al*., 1981; Dawson & Meldrum, 1992) while in blood vessels the story is much less clear. For example, NA content in rat arteries (renal, femoral, and saphenous) increases with age, while in veins (renal, femoral, and saphenous) there is no change, and a decline in tail arteries (Handa & Duckles, 1987). Consistent with NA content in rat arteries, catecholamine histofluorescence, another measure of adrenergic nerve density, increases with age in rat superior mesenteric and renal arteries and portal vein (Mione *et al*., 1988). In contrast, spinal cord blood vessels show no age related change in adrenergic nerve density (Amenta *et al*., 1990). In another study in the internal carotid artery, sympathetic innervation declines with age. However, after intracerebral infusion of NGF, the number of sympathetic axons increases in aged animals (Isaacson & Crutcher, 1998). In a follow-up to this study using internal carotid and anterior cerebral arteries as models, NA content and numbers of tyrosine hydroxylase containing nerve fibres decline with age. However, after infusion of NGF, NA content and the number of tyrosine hydroxylase-containing neurones significantly increased in old animals (Dickason & Isaacson, 2002). These studies would suggest that there is an innate age-related ability to maintain a critical number of functioning sympathetic neurones. The NGF studies cited above offer some interesting possibilities with regards to therapeutic interventions in terms of maintaining cardiovascular homeostasis with advancing age. In light of these studies, there appears to be no clear relationship between age-related changes in adrenergic nerve density, increases in circulating plasma NA levels reported by others and increased stimulation-evoked NA release in our earlier studies. Thus, age-related changes in NA content may be species or vascular bed dependent.

### Transmitter uptake and function of inhibitory prejunctional α_2_-adrenoceptors

Modulation of NA concentration in the synaptic cleft is mediated by a combination of re-uptake and activation of prejunctional α_2_-adrenceptors. The latter mechanism has been shown to attenuate NA release by decreasing stimulation-evoked calcium influx in adrenergic neurones (Schofield, 1990; Delcour & Tsien, 1993). Both mechanisms are critical to controlling the biophase concentrations of NA with ongoing moment-to-moment vascular adrenergic nerve activity (Illes, 1986; Langer & Rbilla, 1990; Buchholz *et al*., 1992; Insel, 1993; Esler *et al*., 1995).

The effect of chemical agents such as cocaine and deoxycorticosterone that block the neuronal and extraneuronal uptake of NA, respectively, is reduced with age in the atria and vas deferens in the pithed rat (Borton & Docherty, 1989; De Avellar *et al*., 1990). These studies suggest that NA uptake declines with age in peripheral adrenergic nerves. Contrasting studies using direct measurement of ^3^H-NA uptake support the idea that the function of NA uptake transporters does not change with age in peripheral adrenergic nerves (Duckles *et al*., 1985). In addition, in the rat heart (Limas, 1975) and tail artery (Buchholz & Duckles, 1990) the effect of cocaine and deoxycoricosterone on NA uptake increased with advancing age. These contrasting studies appear to be difficult to reconcile. However, in the tail artery model, when release of NA in the presence of uptake blockers was corrected for total NA release in the absence of uptake blockers, no change with age was observed (Buchholz & Duckles, 1990). These data suggest that the age-related change in the effectiveness of uptake blockers on NA may reflect the age-related differences in NA concentrations within the junctional cleft of the model under study. In light of the latter approach, overall the data would suggest that NA uptake with advancing age remains as a constant fraction of the amount of NA released.

Taken together, direct measurement of NA uptake and effect of uptake blockers, an age-related increase in stimulation-evoked fractional NA release in peripheral adrenergic nerves is not explained by an age-related change in the function of NA transporters. Thus, age-related changes in NA release may possibly be explained by other mechanisms such as presynaptic inhibition.

Studies measuring NA overflow have shown an age-related decline in the effect of prejunctional α_2_-adrenoceptor antagonists in pithed rats, rat vas deferens and heart and tail artery (Hyland & Docherty, 1985; Docherty & Hyland, 1986; Daly *et al*., 1989; Buchholz & Duckles, 1990; Buchholz *et al*., 1992). These studies appear to support the idea of a general age-related decline in the function of feedback prejunctional α_2_-adrenoceptors. However, we have found that while the sensitivity of the prejunctional α_2_-adrenoceptors to the antagonist, idazoxan, declined with advancing age, there appears to be no age-related difference in the maximal response to this drug (Buchholz *et al*., 1992). Given that fractional stimulation-evoked NA release increases with age, there would be more NA to interact with the α_2_-adrenoceptor and increased competition between higher NA levels and an antagonist. This chemical competition may possibly reduce the apparent sensitivity of the applied antagonist and account for the decrease in the potency of α_2_-adrenoceptor antagonists with age (Pottorf *et al*., 2000b). Overall, in light of others and our studies cited above, it is difficult to explain age-related alterations in NA release in terms of age-related alterations in the function of NA uptake mechanisms or α_2_-adrenoceptor function. Therefore, we looked at other mechanisms that may account for age-related changes in adrenergic nerve activity. These included further studies on stimulation-evoked NA release with altered extracellular calcium, calcium influx and altered calcium buffering capacity.

### Effects of altering extracellular calcium, role of calcium influx and altering buffering capacity on function of peripheral adrenergic nerves

In light of the studies on the tail artery model cited above, our attention was directed toward the possibility that age-related changes in stimulation-evoked NA release is possibly due to changes in calcium regulation in the nerve endings. We carried out experiments to examine the effects of increased or lowered extracellular calcium on stimulation-evoked NA release in tail arteries of young and old rats (Buchholz *et al*., 1994). Stimulation-evoked fractional NA release in tail arteries from old animals was found to be more sensitive to an increase and less sensitive to a decrease in extracellular calcium as compared to young animals. The explanation for these results is complex and may be attributable to altered calcium influx, buffering capacity, or sensitivity of the NA release mechanisms to stimulation-evoked increases in [Ca^2+^]i. Thus, in another study, we measured NA release from rat tail arteries via transmural nerve stimulation or KCl. Under both conditions, NA release was greater in tail arteries from old as compared to young animals (Tsai *et al*., 1997). These data could be explained in part by an age-related increase in calcium influx with age. However, other reports of the impact of age on calcium influx are mixed. Calcium influx has been reported to increase with age in central neurones (Landfield & Pitler, 1984; Pitler & Landfield, 1990), to decrease in peripheral neurones (Kostyuk *et al*., 1993), or to remain unchanged in central neurones (Murchinson & Griffith, 1996). Therefore, in another experiment, we bypassed voltage-gated calcium channels using the calcium ionophore, ionomycin, and found that the age-related increase in NA release persisted (Tsai *et al*., 1997). While stimulation-evoked calcium influx may possibly increase with age in the tail artery model, from the latter experiments we would also argue that other mechanisms such as [Ca^2+^]i buffering and/or alterations in the sensitivity of the NA release mechanisms may also change with age.

We tested the hypothesis that an age-related alteration in [Ca^2+^]i buffering may play a partial role in the increase in NA release from tail arteries (Tsai *et al*., 1997). The addition of the [Ca^2+^]i chelator 1,2-bis(o-aminophenoxy)ethane-N,N,N′,N′-tetraacetic acid (BAPTA) decreased stimulation-evoked NA release to a greater extent in old arteries as compared to young, suggesting that age-related changes in NA release is in part due to altered calcium-buffering capacity. Overall, the age-related changes in the function of peripheral adrenergic neurones in terms of NA release appears to be at least due in part to altered [Ca^2+^]i handling mechanisms. However, altered sensitivity of the NA release mechanisms has not been ruled out.

## Alterations in neuronal calcium buffering and extrusion during aging in peripheral neurones

The introduction of cell permeant fluorometric calcium indicator dyes such as Fura-2, allowed for the measurement of real-time changes in [Ca^2+^]i in living cells (Tsien *et al*., 1985; Roe *et al*., 1990; Thayer & Miller, 1990; Neher, 1995). Microfluorometry coupled with calcium sensitive dyes allows investigators to measure the impact of advancing age on the mechanisms that modulate stimulation-evoked [Ca^2+^]i transients and overall calcium homeostasis (Kirischuk *et al*., 1992; Kirischuk & Verkhratsky, 1996; Neher, 1998; Baylor & Hollingworth, 2000; Pottorf *et al*., 2002).

Each component of the [Ca^2+^]i buffering and extrusion system participates in controlling the shape and duration of stimulation-evoked [Ca^2+^]i transients (Fig. 1A). An age-related alteration in any one or a combination of the components of this [Ca^2+^]i control system may alter the function of neurones and or contribute to neuronal degeneration (Meldrum & Garthwait, 1990; Peterson, 1992; Verkhratsky *et al*., 1994; Pottorf *et al*., 2000b, 2002). Given the complex interplay between numerous buffering systems to control [Ca^2+^]i transients, declining function of one or more systems may be compensated for by increased function of other mechanisms (Pottorf *et al*., 2000b).

### Age-related decline in SERCA function in peripheral neurones

Active transport of calcium into the SER is mediated by SERCA and an age-related decline in their function has been suggested to contribute to calcium dysregulation in peripheral sympathetic and sensory neurones (Kirischuk *et al*., 1992; Kirischuk & Verkhratsky, 1996; Pottorf *et al*., 2002). In addition, a decline in SERCA function with age may have greater consequences. For example, SERCA buffer [Ca^2+^]i transients and load the SER calcium stores, thus, an age-related decline in their function may possibly cause alterations in CICR from the SER (Murchinson & Griffith, 1999; Usachev & Thayer, 1999a).

In our studies in adrenergic nerve endings in tail arteries, the SERCA blocker, thapsigargin, increased stimulation-evoked NA release in young arteries with no change in arteries from old animals (Tsai *et al*., 1998). In isolated SCG cells the SERCA blocker cyclopiazonic acid or thapsigargin caused a decline in the rate of recovery of high K^+^-evoked [Ca^2+^]i transients only in cells from young animals with no significant change in old cells (Tsai *et al*., 1998). In another study using SCG cells, we blocked the contribution of PMCA's, mitochondrial calcium uptake, and the Na^+^/Ca^2+^ exchanger in controlling high K^+^-evoked [Ca^2+^]i transients. Under these conditions, the neurones are required to rely on SERCA to modulate the rate of recovery of high K^+^-evoked transients, and clearly the rate of recovery was slower in old as compared to young SCG neurones (Pottorf *et al*., 2000c). Overall, one mechanism that may account for an age-related decline in [Ca^2+^]i homeostasis in peripheral neurones is a decline in SERCA function. A reasonable explanation for a decline in SERCA function is that there is a decline in the genetic expression of SERCA isoforms and this has been shown in cardiac tissue (Maciel *et al*., 1990). However, in skeletal muscle and myocardium SERCA-mediated ^45^Ca^2+^ uptake declines with age while SERCA protein levels remain stable. These data suggest that the change in function of SERCAs is possibly mediated by a decline in their modulation by mechanisms such as phosphorylation, as opposed to a decline in protein levels *per se*. (Gafni & Yuh, 1989; Xu & Narayanan, 1998). Further studies are necessary to determine the mechanisms that account for why SERCA function is altered with age.

### Aging and mitochondrial calcium uptake in peripheral neurones

Mitochondrial calcium uptake has been shown to be a robust mechanism in the control and shaping of [Ca^2+^]i transients in peripheral and central neurones (Thayer & Miller, 1990; Buchholz *et al*., 1996; Murchinson & Griffith, 1999; Colegrove *et al*., 2000). In addition, it has been suggested that an age-related decline in mitochondrial function may be a mechanism that promotes cellular aging (Duckles *et al*., 2006). As mitochondria participate in calcium buffering, if there is a decrease in mitochondrial function with age, or if there is an increased reliance on mitochondria to control [Ca^2+^]i levels, both conditions may possibly result in disruption of mitochondrial function due to calcium overload and apoptosis. There are studies in central neurones supporting an age-related decline in mitochondrial calcium uptake (Vitorica & Satrustegui, 1985; Villalba *et al*., 1995; Satrustegui *et al*., 1996). However, our studies in isolated SCG neurones and adrenergic nerve endings suggest that mitochondrial calcium uptake in peripheral neurones may be preserved with age. For example, when SCG neurones are exposed to dinitrophenol to block mitochondrial calcium uptake, peak, and rate of rise of high K^+^-evoked [Ca^2+^]i were only increased in neurones from old animals with no significant effect in young neurones (Buchholz *et al*., 1996). Similarly, in adrenergic nerve endings in tail arteries, dinitrophenol increased stimulation-evoked NA release in arteries from old animals with no significant effect in young arteries (Tsai *et al*., 1995). In another study, we blocked PMCA, SERCA, and the Na^+^/Ca^2+^ exchanger in SCG neurones to cause the neurones to rely primarily on mitochondrial calcium uptake to regulate [Ca^2+^]i transients. The results showed that the capacity of mitochondrial calcium uptake did not change with age (Pottorf *et al*., 2000a). What is most interesting about these studies is that when multiple calcium modulatory systems are blocked, mitochondria are still capable of controlling [Ca^2+^]i transients in SCG neurones and appear to be preserved with age. This increased reliance on mitochondria to control [Ca^2+^]i may place an added stress on aged neurones. For example, in brain slices from mice, mitochondrial depolarization as an index of mitochondrial calcium uptake in response to high K^+^-evoked [Ca^2+^]i transients, occurs in both young and old mice. However, in the old animals the rate of mitochondrial repolarization is slower that corresponded to slowed recovery of high K^+^-evoked [Ca^2+^]i transients (Xiong *et al*., 2002). Thus, in the case of high neuronal activity in CNS neurones, the ability of the mitochondria to sustain its buffering of repeated [Ca^2+^]i transients may be compromised.

The studies cited above are consistent with other studies in CNS neurones suggesting that mitochondrial calcium uptake is essential in the modulation of [Ca^2+^]i transients with advancing age (Murchinson & Griffith, 1998, 1999). In a more recent review on CNS neurones, it has been suggested that as more stress is placed on the mitochondria to control stimulation-evoked increases in [Ca^2+^]i the ‘polarization state’ of the mitochondria may subtly decline over the lifespan (Toescu, 2005). This would in turn lead to a gradual decline in function of the mitochondria in terms of controlling [Ca^2+^]i and possibly maintaining energy status of the cell. Overall, the cited studies argue that mitochondrial calcium uptake may become more central to controlling [Ca^2+^]i transients in neurones with advancing age. In addition, maintaining mitochondrial function with improved diet and exercise as we age is emerging as an important topic in relation to cellular aging (Duckles *et al*., 2006).

### Aging and function of plasma membrane calcium transport systems

The PMCA and Na^+^/Ca^2+^ exchange systems are two plasma membrane calcium-pumping mechanisms in peripheral and central neurones that play an important role in buffering [Ca^2+^]i transients via extrusion of calcium from the cytosol (Werth *et al*., 1996; Blaustein & Lederer, 1999; Pottorf & Thayer, 2002; Pottorf *et al*., 2006). Blockade of SERCA and prolonged calcium transients have been shown to induce PMCA gene expression and accelerate the function of PMCA via increased interaction of calmodulin with PMCA (Kuo *et al*., 1997; Pottorf & Thayer, 2002). These data suggest that components of the calcium buffering systems can compensate for a reduction in function of one component or in the case of prolonged [Ca^2+^]i transients. Indeed, in the failing myocardium, the Na^+^/Ca^2+^ exchanger levels increase as SERCA levels decline (Hasenfuss *et al*., 1991).

Our studies in isolated SCG neurones support the notion that multiple components of the calcium buffering systems can compensate for a loss of SERCA function. Indeed, we have found that SCG neurones become more reliant on mitochondria to control [Ca^2+^]i transients. When SERCA and mitochondrial calcium uptake were blocked in SCG neurones, the PMCA and Na^+^/Ca^2+^ exchanger are required to control high K^+^-evoked [Ca^2+^]i transients. Under these experimental conditions, SCG neurones from both young and old animals were able to fully recover from high K^+^-evoked [Ca^2+^]i transients (Tsai *et al*., 1998). These data suggest that in peripheral adrenergic neurones plasma membrane calcium extrusion systems can by themselves control [Ca^2+^]i and their function is maintained with advancing age. In support of this study, we used vanadate, which at low concentrations (0.25 µm) blocks PMCA function but does not significantly affect SERCA function. Under these conditions, the rate of recovery was diminished to a greater extent in SCG neurones from old as compared to young animals (Pottorf *et al*., 2000a). These data suggest that PMCA function may increase with advancing age as SERCA function declines. Although PMCA function has been reported to decline with age in synaptosomes derived from neurones in the CNS (Qin *et al*., 1998), our studies suggest that during normal aging PMCA function in peripheral adrenergic neurones is maintained with age.

### Model for age-related changes in [Ca^2+^]i regulation in peripheral adrenergic neurones

One hypothesis suggested by numerous researchers is that advancing age leads to [Ca^2+^]i dysregulation and neuronal loss (Choi, 1992; Kirischuk & Verkhratsky, 1996; Ichas & Mazat, 1998; Thibault *et al*., 1998; Begley *et al*., 1999). However, this hypothesis may apply more to pathological conditions as opposed to normal aging. Thus, in light of our studies of peripheral adrenergic neurones we pose another hypothesis. That is advancing age in the absence of pathology results in subtle changes in the control of [Ca^2+^]i that may lead to altered neuronal function, and that other [Ca^2+^]i control mechanisms may allow neurones to adapt to an age-related decline in the control of [Ca^2+^]i. This model is summarized in Fig. 1(B) and emphasizes that loss of SERCA function may be compensated for by increased buffering by mitochondria and plasma membrane calcium extrusion so as to preserve some degree of cell viability in the face of advancing age.

## Consequences of age-related decline in SERCA Function in peripheral adrenergic neurones: CICR

The function of neurones depends in part on the release of calcium from the SER in response to an elevation in [Ca^2+^]i mediated by voltage-gated Ca^2+^ channels (Usachev & Thayer, 1997, 1999a,b). This process has been termed CICR and is relevant in processes such as release of neurotransmitters and hormones.

To sustain calcium release during neuronal activity requires refilling of the SER calcium through calcium influx and subsequent uptake into the SER via SERCA pumps (Kostyuk & Verkhratsky, 1994; Verkhratsky *et al*., 1994; Shmigol *et al*., 1996). Thus, buffering of [Ca^2+^]i transients and refilling [Ca^2+^]i stores by SERCA suggest that calcium release and [Ca^2+^]i buffering are intimately related processes. In CNS, SCG, and sensory neurones, SER Ca^2+^ stores can be rapidly refilled by activation of voltage gated calcium channels with high K^+^ or they can spontaneously refill within 3–10 min following depletion with caffeine via activation of store operated calcium channels (Friel & Tsien, 1992; Shmigol *et al*., 1994, 1996; Usachev & Thayer, 1999b; Baba *et al*., 2003).

In the discussion above, our work has consistently shown that SERCA mediated calcium uptake declines with age in isolated SCG neurones. Given that SERCA function declines with age in the SCG we studied how aging may alter the refilling and release of Ca^2+^ from the SER. We measured both rapid depolarization evoked refilling of SER calcium stores and spontaneous refilling following caffeine-evoked depletion of SER calcium stores in isolated Fura-2 loaded SCG neurones from rats aged 6, 12, 20, and 24 months. The data showed that both rapid and spontaneous refilling of SER via SERCA declined with age. Overall, the data suggest that a functional consequence of reduced SERCA activity with advancing age is a compromise in the ability of SCG neurones to sustain release of [Ca^2+^]i during ongoing neuronal activity (Vanterpool *et al*., 2005).

## Altered expression of ryanodine receptors and selective modulators of CICR with advancing age

Calcium-induced calcium release is mediated via the RyR channels and their function depends in part on density and regulation. The regulation of the function of RYRs is complex and overall this regulation serves to modulate the sensitivity of RYRs to cellular Ca^2+^ levels. These modulators include FK506 binding protein proteins, which serve to activate or inhibit channel state depending on its binding status, and activators such as phosphorylation and intracellular molecules including cyclic adenosine diphosphate ribose (cADPr) (Hua *et al*., 1994; Ogawa *et al*., 2000; Marx & Marks, 2002). Specifically, cADPr levels are modulated by nitric oxide released from nNOS containing neurones.

As RYRs are integral components in [Ca^2+^]i signaling in SCG neurones, we used molecular techniques of reverse transcription-polymerase chain reaction and enzyme-linked immunosorbent assay to test the hypothesis that the genetic and protein expression of the predominant RyR isoform(s) in adult rat SCG, along with selective modulators, are altered during late maturation and advancing age in F-344 rats aged 6, 12, and 24 months (Vanterpool *et al*., 2006).

Surprisingly, we have found that RyR1 mRNA was undetectable in the rat SCG, in contrast with other studies demonstrating RyR1 mRNA is expressed in excitable cells, including neurones (Fill & Copello, 2002). Thus, independent of late maturation and advancing age RyR1 does not appear to play a role in mediating the release of calcium in the SCG. However, RyR2 and RyR3 are the major receptor isoforms that regulate calcium release from RyR-sensitive stores in the SCG in all age groups. In addition, during late maturation from 6 to 12 months RyR3 mRNA and protein levels increased and then decreased in senescent (24 month) animals while RyR2 mRNA and protein levels remained constant during late maturation and advancing age (Vanterpool *et al*., 2006). It is difficult to make a straightforward conclusion as to the functional consequences of this age-related decline in RyR levels. We have shown that caffeine-evoked release of calcium declines with age (Vanterpool *et al*., 2005) and the capacity to release calcium from the SER depends on SER calcium filling levels, as well as the modulation of the RYRs. Indeed, we have shown that SERCA function declines in the SCG (Tsai *et al*., 1998; Pottorf *et al*., 2000c). Thus, filling levels of the SER may also be compromised that may alter the functional capacity of the release mechanism. In addition, It has been reported that RyR function can be influenced by several factors, including phosphorylation, binding proteins, calcium levels, and nNOS, which modulates cADPr levels that in turn modulates CICR by changing the sensitivity of RYRs to changes in [Ca^2+^]i (reviewed in Galione, 1993; Eu *et al*., 1999; Balshaw *et al*., 2002; Meissner, 2002; Danila & Hamilton, 2004).

In addition to measuring the impact of age on RyR gene expression and protein levels, we extended our studies to include possible age-related changes in selected modulators of the RYRs, which include phosphorylation and nNOS levels within the SCG (Vanterpool *et al*., 2006). Total phosphorylation of RyR channels was not altered with age suggesting that changes in steady state phosphorylation and hence regulation by this mechanism is not necessarily occurring. However, nNOS protein expression increases from 6 to 12 months and significantly declines from 12 to 24 months. As nNOS activity modulates cADPr levels, it is reasonable to speculate that these data may possibly suggest that cADPr levels may also decline with age. As there appear to only be two RYRs contributing to calcium release in the SCG, overall, given the age-related decline in RyR3, coupled with a decline in nNOS levels, we hypothesize that an age-related reduction in RyR3 receptor levels and cADPr levels may account in part for a decline in the function of RYRs. We are currently determining if advancing age alters cADPR levels in the SCG, which may shed light on activity of nNOS during the aging process and regulation of RYRs.

## Summary and conclusions

The data suggest that normal aging in peripheral autonomic neurones is a subtle and complex process and does not always result in dramatic deterioration. Moreover, the aging process does not necessarily alter the function of excitable neurones in a uniform manner. In terms of age-related changes in [Ca^2+^]i regulation, we present the idea that in order to maintain cell viability peripheral neurones are able to compensate for an age-related decline in the function of at least one calcium-buffering system, SERCA, by increased function of other calcium-buffering systems, namely, the mitochondria and plasmalemma calcium extrusion. Increased mitochondrial calcium uptake may represent a ‘weak point’ in cellular compensation as this over time may contribute to cell death (Ichas & Mazat, 1998; Thibault *et al*., 1998; Begley *et al*., 1999).

**Fig. 2:**
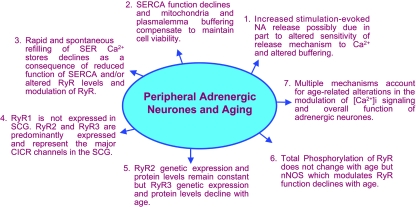
Summary of major findings of age-related alterations in the function of peripheral autonomic neurones and [Ca^2+^]i regulation.

This review summarizes our work on the dynamics of intracellular calcium regulation and possible consequences for autonomic nerve function with advancing age. The major findings of all of our studies are summarized in Fig. 2. Based on results of our most current studies (Vanterpool *et al*., 2005, 2006) and our previous work and that of others we propose that an age-related alteration in [Ca^2+^]i signaling and function of peripheral adrenergic neurones represents a complex interplay of mechanisms, including increased sensitivity of the neurotransmitter release mechanism to calcium, a decline in SERCA function that alters calcium buffering and refilling of SER calcium stores, reduced RyR3, and nNOS levels, which in turn modulate cADPr levels and the CICR process. The consequences of these changes are currently being studied in our laboratory by direct measurement of cADPr during aging and the contribution of release of calcium from SER stores to stimulation-evoked increases in [Ca^2+^]i. Given the advances in molecular techniques future studies may include a comprehensive study on the impact of age on the genetic expression and protein levels of multiple buffering systems, including soluble calcium-buffering proteins, SERCA, mitochondrial calcium pumps, PMCA, and the Na^+^/Ca^2+^ exchanger. In addition, another mechanism that modulates calcium influx and SER refilling are store operated calcium channels. Their genetic expression and function with advancing age has not been studied and may provide a fruitful avenue of future research. Finally, SERCA function declines with age thus possibly altering the levels of SER calcium stores and CICR. With calcium indicators available to study SER calcium levels, it is possible to examine how advancing age may impact the calcium levels of the SER. This type of study may provide additional insight on how CICR may be altered with advancing age.
